# Relationship between the change in infrapatellar fat pad thickness assessed using ultrasonography and anterior knee pain on squatting after anterior cruciate ligament reconstruction

**DOI:** 10.1007/s10396-023-01300-3

**Published:** 2023-03-24

**Authors:** Ryo Shiraishi, Shinichiro Ueda

**Affiliations:** 1 Rokuto Orthopedic Surgery Az, 46 Onoyama-cho, Naha City, Okinawa 900-0026 Japan; 2 Department of Clinical Research and Quality Management, Graduate School of Medicine, University of The Ryukyus, 207 Uehara, Nishihara-cho, Okinawa, 903-0215 Japan; 3 Present Address: Department of Rehabilitation Therapy, Chuzan Hospital, 6-2-1 Matsumoto, Okinawa City, Okinawa 904-2151 Japan

**Keywords:** Anterior cruciate ligament reconstruction, Ultrasonography, Infrapatellar fat pad

## Abstract

**Purpose:**

Anterior knee pain (AKP) may occur after anterior cruciate ligament (ACL) reconstruction. The present study investigated the relationship between the change in infrapatellar fat pad (IFP) thickness assessed using ultrasonography (US) and AKP on squatting in patients after ACL reconstruction.

**Methods:**

Patients were enrolled 3 months after ACL reconstruction using the bone-tendon-bone (BTB) technique. Subjects were divided into the AKP group (numerical rating scale [NRS] score ≥ 1) and control group (NRS score < 1) using a NRS of pain on squatting, and intergroup comparisons were performed. On US evaluation, measurement angles of the knee joint were 0° and 30° in the supine position. The IFP between the femoral intercondylar notch and patellar tendon was measured on short-axis images. The changes in IFP thickness were calculated from values measured at different angles of the knee joint.

**Results:**

Twenty-one patients (mean age 24.9 ± 9.3 years) were included in the present study: 12 in the AKP group (9 males, 3 females) and nine in the control group (5 males, 4 females). A significant difference in the change in IFP thickness at 3 months was observed between the AKP and control groups (0.67 ± 0.44 mm vs. 1.84 ± 0.34 mm, p < 0.001). There was a negative correlation between the change in IFP thickness and the NRS score (r = − 0.720, p < 0.001) in reconstructed knees.

**Conclusion:**

A smaller change in IFP thickness assessed using US after ACL reconstruction was identified as a factor contributing to AKP on squatting.

## Introduction

Anterior cruciate ligament (ACL) injuries are the most common knee joint injuries. Anterior knee pain (AKP) is a complication of ACL reconstruction [1, 2]. AKP has been shown to decrease activities of daily living (ADL) and sports activities [3, 4]. Niki et al. reported on the incidence of AKP in patients who underwent ACL reconstruction using the bone-tendon-bone (BTB) technique and found that 42% of patients developed AKP within 3 months after surgery [3]. Kovindha et al. reported a 62.9% incidence of AKP at 3 months after surgery. Thus, these findings suggest that many patients develop AKP within 3 months after ACL reconstruction [5]. Biau et al. state that one of the main reasons for the high incidence of AKP with the BTB technique is that reconstructive surgery with the BTB technique is one of the main factors causing the symptoms of anterior knee pain [6]. In addition, standard ACL reconstruction is often performed with a central one-third patellar tendon graft because of the superior strength of the patellar tendon graft and the ability to anchor the graft via patellar and tibial bone blocks. Therefore, a patellar tendon defect is created with the removal of the central one-third of the patellar tendon; closure of that defect can cause lowering of the patella and can consequently lead to increased sensitivity and pain when the anterior compartment is directly pressed during kneeling or squatting.

The infrapatellar fat pad (IFP) has been identified as a factor contributing to AKP after ACL reconstruction [7]. A previous study evaluated the dynamics of the IFP using ultrasonography (US) after ACL reconstruction in patients who had undergone semitendinosus-gracilis (STG) procedures [8]. A decreased ratio of change in IFP measured with magnetic resonance imaging (MRI) and US had a negative impact on pain and lower extremity motor function in deep flexion [8, 9]. Another study that examined AKP using US reported a decreased ratio of change in IFP on the reconstructed side after ACL reconstruction via the STG technique, which was associated with AKP [10].

Many patients are rehabilitated to sports activities after ACL reconstruction. Squatting, an exercise performed to improve lower extremity motor function, is a safe and efficient lower limb exercise even early after ACL reconstruction [11]. However, AKP has been reported during lower extremity exercises such as squatting [12], and the IFP has been identified as one of the contributing factors [7]. For an exercise such as squatting, it is possible that the IFP may change shape as the knee joint moves. Some studies in healthy individuals suggest that the IFP moves due to the possibility of its migrating from postero-superior to anterior as the knee joint moves [13]. However, synovial fibrosis of the IFP occurs after ACL reconstruction. In addition, synovial fibrosis of the IFP limits shape changes, inducing further mechanical stress on the IFP and causing AKP.

Although IFP dynamics have been investigated after ACL reconstruction, the relationship between squatting and IFP dynamics assessed using US after ACL reconstruction in patients who undergo the BTB technique remains unknown. The relationship between IFP dynamics assessed via US and squatting warrants further study from the perspective of improving ADL and sports activities. This study aimed to investigate the relationship between AKP during squatting and the dynamics of the IFP as assessed with US after ACL reconstruction using the BTB technique.

## Materials and methods

**Fig. 1:**
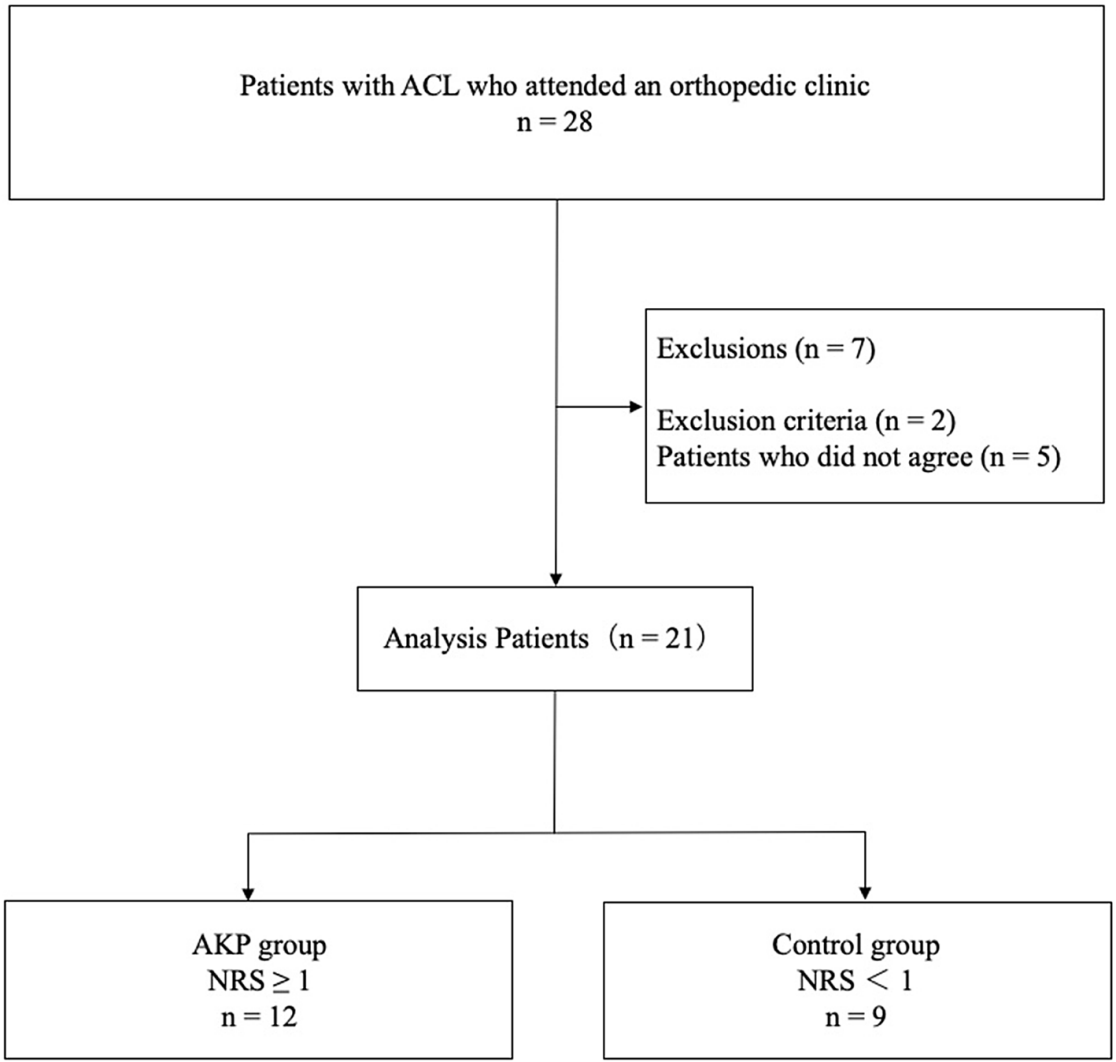
Flowchart of the study population. *ACL* anterior cruciate ligament, *AKP* anterior knee pain, *NRS* numerical rating scale

Of 28 patients who underwent ACL reconstruction between November 2018 and December 2019 for the resumption of ADL and sports activities, US was performed for 21 patients who gave their written informed consent to this study. Parental consent was obtained for subjects younger than 20 years of age. US evaluations were performed 3 months after ACL reconstruction. Patients with multiple ligamentous injuries and bilateral ACL injuries and those who did not provide consent were excluded (Fig. 1). All reconstructive procedures were performed arthroscopically by our orthopedic surgeons according to the surgical technique in previous studies [14]. All patients underwent ACL reconstruction using the BTB technique. The graft used for the reconstruction procedure was the patellar tendon, and the graft was made to have a diameter of 10 mm. In addition, a portion of the IFP was resected to provide a clear field of view during arthroscopic surgery. After ACL reconstruction, all patients underwent the same rehabilitation protocol. Patients were allowed knee joint flexion of 130° after ACL reconstruction by the fifth week. At 3 months after surgery, the patients were allowed to jog, and wearing a knee brace for 4 months after surgery was recommended. All patients had a knee flexion angle of approximately 140° by 3 months after surgery. The extension angle was 0° in all patients. The range of motion of the knee joint of all patients was obtained from active measurements.

### Data collection

Age, sex, height, weight, and body mass index (BMI) were collected from medical records.

### US evaluation

**Fig. 2:**
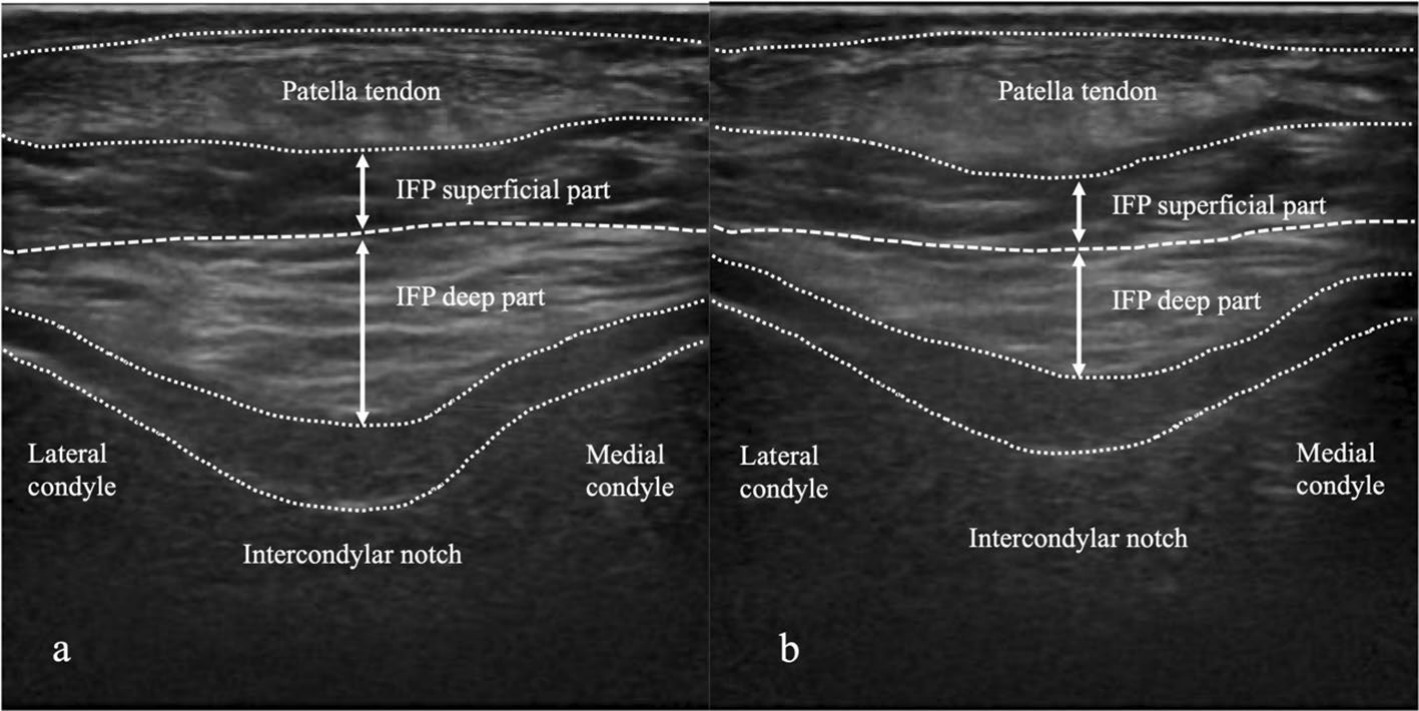
Ultrasonography image. The thickness of the superficial and deep part of the IFP was measured at 30° of flexion (**a**) and 0° of extension (**b**) of the knee joint. IFP, infrapatellar fat pad

**Fig. 3:**
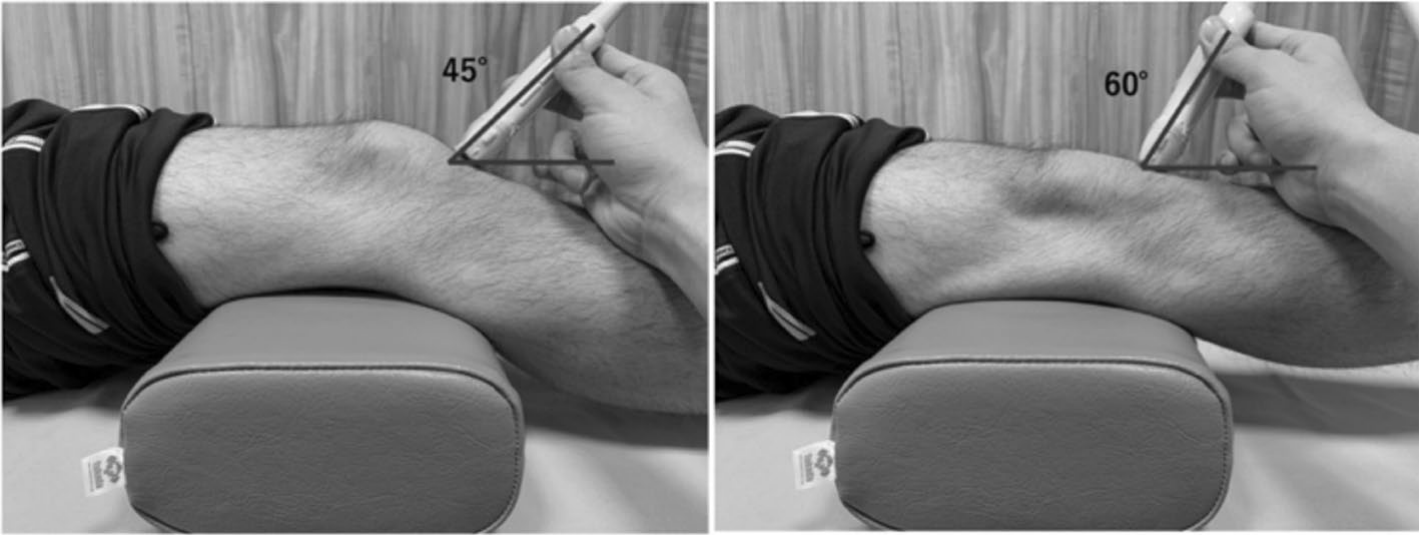
Photograph showing how the ultrasonography image is captured. An angle of incidence of 60° is used for the 0° knee joint and 45° for the 30° knee joint

US was used to evaluate the IFP. In this study, the HITACHI digital ultrasound system Noblus (ALOKA, Hitachi Medical Corporation, Kashiwa City, Japan) was used. A linear transducer (5–10 MHz) was used, and the image display mode was the B mode. The US evaluation was performed on both the ACL-reconstructed knee and the non-reconstructed knee. IFP thickness was measured on short-axis images using the femoral intercondylar notch and patellar tendon as landmarks so that each landmark was clearly delineated (Fig. 2) [15]. IFP thickness was measured in the superficial and deep part of the thickest area at the midpoint between the medial and lateral condylar. Measurements were summed for the surface and deep parts. IFP thickness was measured using a digital measuring tape on the ultrasound system. The limb position was supine during measurement, and the knee joint measurement angles were set at 0° and 30° according to previous studies [13]. The standard position for the measurement method was slight flexion of the knee joint. The knee joint angle was measured using a goniometer, and a pillow was placed under the knee to maintain the joint angle. The angle of incidence of the transducer was standardized using a goniometer according to the angle of the knee joint. An angle of incidence of 60° was used for the 0° knee joint and 45° for the 30° knee joint (Fig. 3). Three measurements were performed at each limb position, and the average value was used in the analysis. In addition, US images were also measured by two examiners trained in US evaluation. The changes in IFP thickness were calculated from the values measured at different angles of the knee joint (Change in thickness values = value at the 30° knee joint angle—value at the 0° knee joint angle).

### AKP evaluation and definition of patients

AKP on squatting was evaluated using a numerical rating scale (NRS). Patients with an NSR score of ≥ 1 point were classified as the AKP group, with the others as the control group. In addition, patients were evaluated for pain 3 months after ACL reconstruction.

### Statistical analysis

All continuous variables were checked for normality with the Kolmogorov–Smirnov test. Age, height, weight, BMI, NRS, IFP thickness value, and change in IFP thickness were compared using the t-test or the Mann–Whitney U test. Fisher’s exact test was used for sex comparisons between the AKP and control groups. In addition, Spearman’s rank correlation coefficient was used to examine the correlation between the change in IFP thickness and the NRS score on the reconstructed knee. Quantitative variables are expressed as means ± standard deviations and qualitative variables as frequencies. JMP^®^ Pro Ver. 15 (SAS) was used for all analyses, and the significance level was set at < 5%.

## Results

**Fig. 4:**
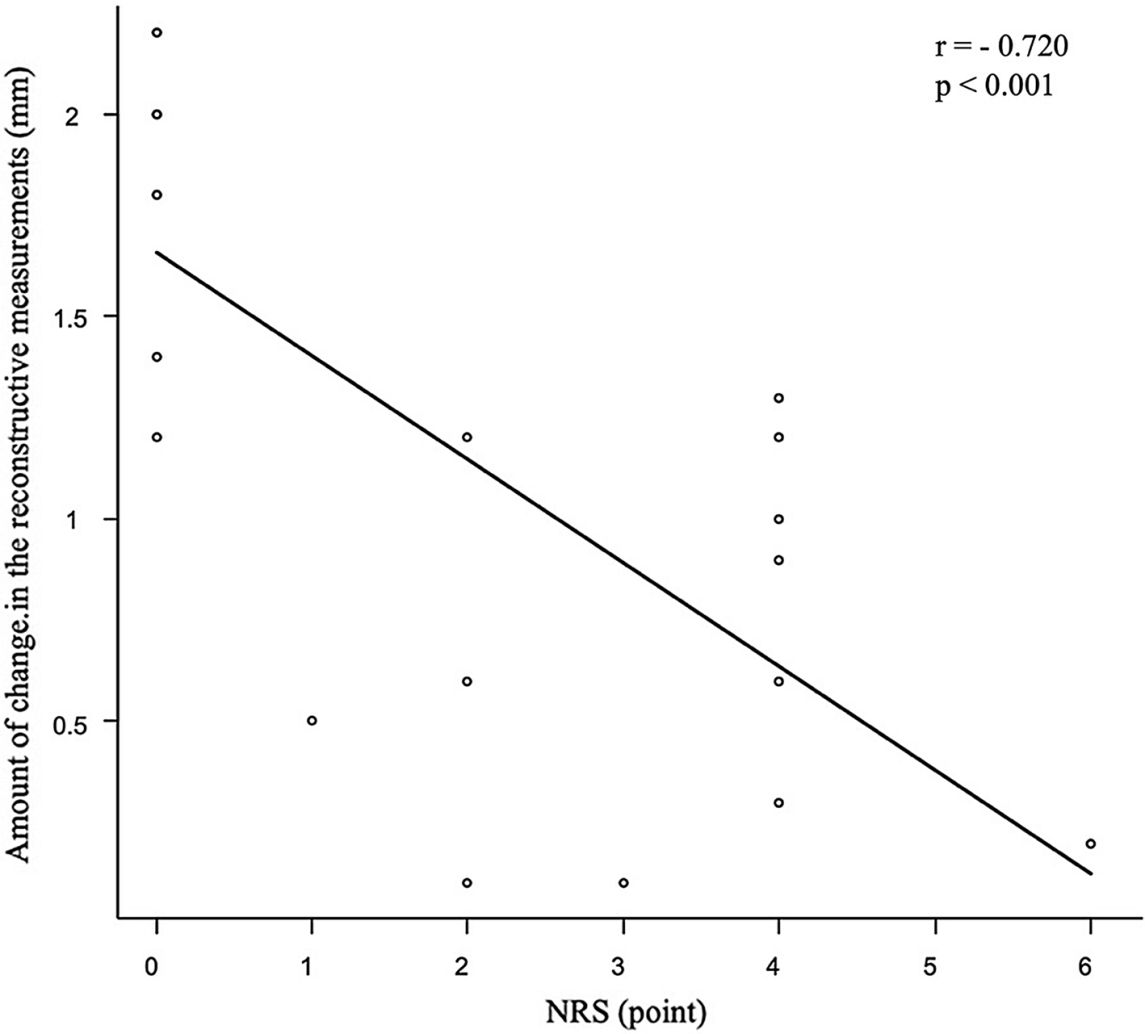
Correlation between the amount of change in IFP thickness and the NRS score on the reconstructed knee. *IFP* infrapatellar fat pad, *NRS* numerical rating scale

Twenty-one patients (14 males and 7 females; mean age 24.9 ± 9.3 years) who provided consent to participate in this study were included in the analysis; there were 12 in the AKP group (9 males and 3 females) and nine in the control group (5 males and 4 females) (Table 1). IFP thickness values and the change in IFP thickness after 3 months are shown in Table 2. Of the reconstructive surgery measurements, a significant difference was observed in the change in IFP thickness between the AKP and control groups (0.67 ± 0.44 vs. 1.84 ± 0.34 mm, *p* < 0.001). No significant difference was noted in IFP thickness values between the non-reconstructed and reconstructed knee measurements. Figure 4 shows the correlation between the change in IFP thickness and the NRS score on the reconstructed knee of both groups. There was a negative correlation between the change in IFP thickness and the NRS score in reconstructed knee measurements (r = − 0.720, p < 0.001). A smaller change in IFP was negatively correlated with the intensity of pain.

**Table 1:** Patient’s demographic characteristics

	Overall	AKP group	Control group	*p* value
	(*n* = 21)	(*n* = 12)	(*n* = 9)	
Age (year)	24.9 ± 9.3	25.7 ± 10.23	23.9 ± 8.34	0.675
Sex (male: female)	14 (67): 7 (33)	9 (75): 3 (25)	5 (56): 4 (44)	0.397
Height (cm)	167.5 ± 7.6	169.2 ± 7.0	165.4 ± 8.2	0.267
Body weight (kg)	63.6 ± 9.1	63.4 ± 8.2	63.8 ± 10.7	0.930
BMI (kg/m^2^)	22.6 ± 2.6	22.1 ± 2.3	23.2 ± 2.9	0.326

Continuous variables are shown as means ± standard deviation (%)

*BMI* body mass index

**Table 2:** IFP thickness values and the change in IFP thickness after 3 months

	Overall	AKP group	Control group	*p* value
	(*n* = 21)	(*n* = 12)	(*n* = 9)	
NRS, points	1.9 ± 2.0	3.3 ± 1.4	0	< 0.001
IFP thickness values				
Non-reconstructed knee measurements				
Flexion (mm)	16.25 ± 2.00	15.95 ± 1.86	16.66 ± 2.22	0.982
Extension (mm)	13.83 ± 1.89	13.84 ± 1.87	13.82 ± 2.04	0.437
Reconstructed knee measurements				
Flexion (mm)	14.84 ± 2.63	14.05 ± 2.80	15.90 ± 2.09	0.113
Extension (mm)	13.67 ± 2.50	13.38 ± 2.90	14.06 ± 1.97	0.557
Change in the non-reconstructed knee measurements (mm)	2.41 ± 1.01	2.11 ± 0.73	2.83 ± 1.23	0.106
Change in the reconstructed knee measurements (mm)	1.17 ± 0.71	0.67 ± 0.44	1.84 ± 0.34	< 0.001

*NRS* numerical rating scale, *IFP* infrapatellar fat pad

The validity of the measurement method was calculated from the intra-rater correlation coefficient (ICC; 1.1), which was 0.89, and the inter-rater correlation coefficient (ICC; 2.1), which was 0.98.

## Discussion

This study examined the relationship between the changes in IFP thickness assessed using US and AKP after ACL reconstruction performed using the BTB technique. The results showed that a smaller change in IFP thickness was associated with AKP. Furthermore, there was a negative correlation between the changes in IFP thickness on the ACL-reconstructed knee and AKP pain intensity.

A decrease in the change in IFP thickness after ACL reconstruction was a factor contributing to AKP on squatting. After ACL reconstruction, the volume of IFP assessed using MRI was previously reported to be lower on the reconstructed side than on the non-reconstructed side [9]. In addition, a study that evaluated the ratio of change in and volume of IFP thickness using US in patients who underwent ACL reconstruction via the STG technique reported that a decrease in this ratio and volume had a negative impact on deep flexion and lower limb motor function [8, 9]. Another study that examined the ratio of change in IFP thickness using US identified a decrease in this ratio after reconstruction with the STG technique as a factor contributing to AKP [10]. Some studies have shown that synovial fibrosis of the IFP occurs after ACL reconstruction and after arthroscopy [7, 16]. In addition, synovial fibrosis of the IFP limits shape changes, inducing further mechanical stress on the IFP and causing AKP. Therefore, a decrease in the change in IFP thickness after ACL reconstruction performed using the BTB technique may limit IFP dynamics in the knee joint, resulting in AKP on squatting.

IFP thickness has been evaluated with US in studies of patients who underwent ACL reconstruction and those with knee osteoarthritis, and its utility has been demonstrated [8, 17]. This study included patients who had undergone ACL reconstruction using the BTB technique; however, previous studies showed that the incidence of AKP after ACL reconstruction was significantly higher after the BTB technique than after the STG technique [1, 2]. Although the IFP has been quantitatively evaluated by means of MRI and magnetic resonance elastography [7, 18], these methods are rarely used in clinical practice because of the associated time and expense. Therefore, the results of the present study, which showed an association between IFP dynamics assessed using US and AKP, demonstrate the usefulness of assessing IFP thickness using US in post-ACL reconstruction patients with AKP. This may be an important assessment for resumption of ADL and sports activities [9]. This study is the first to show an association between IFP dynamics and AKP after ACL reconstruction using the BTB technique. The results showed that the change in IFP thickness was lower in patients who had AKP after ACL reconstruction. Furthermore, a smaller IFP change on the ACL reconstruction side was negatively correlated with pain during squatting, and pain tended to increase with a smaller change in IFP. These results suggest that, in patients with AKP during squatting after ACL reconstruction, IFP dynamics should be evaluated using US, rather than assessing only pain. In addition, real-time information may be obtained in the clinical setting by using US to evaluate IFP movement against AKP during squatting, which may provide a better diagnostic approach. Furthermore, the information gained from the noninvasive US evaluation of AKP after ACL reconstruction can be shared with the patient. The results of the present study may help to evaluate IFP dynamics after ACL reconstruction with the BTB technique.

Several limitations need to be addressed. Only patients who gave consent to participate in the study were included. Furthermore, the sample size was small and, thus, cannot be generalized to all patients who undergo ACL reconstruction. In addition, the measurement period was only 3 months postoperatively. Therefore, a prospective study longer than 3 months is needed in the future. In this study, the dynamics of IFP were evaluated at different angles of the knee joint. However, when the knee joint is in extension, the contraction of the quadriceps muscle likely affects IFP values measured. Therefore, it could be that it was difficult to remove the variation in the acquired measurements. Future study designs should consider the effects of quadriceps muscle contraction. When reconstructive surgery is performed using the BTB technique, some of the IFP may be resected to maintain the field of view [19]. Therefore, the ablative volume of the IFP may have affected the results obtained. A previous study suggested that IFP blood flow was a factor contributing to AKP, and, thus, qualitative and quantitative changes need to be examined [20]. In addition, the US evaluation of IFP dynamics was not performed under weight-bearing conditions [18]. Therefore, caution should be exercised in interpreting the results of this study. Traditionally, it has been reported that AKP after ACL reconstruction occurs in the active weight-bearing condition. In this study, the dynamics of IFP were not evaluated under weight-bearing conditions, so the method of measurement differed from the conditions under which AKP occurs. Therefore, the dynamics of IFP under non-weight-bearing conditions alone cannot be used to refer to a causal relationship with AKP. However, previous studies have reported that changes in IFP kinetics evaluated under non-weight-bearing conditions were associated with AKP [21]. Therefore, the results of this study also suggest that the change in IFP thickness as assessed using US is a factor in AKP. Since AKP often adversely affects ADL and sports activities, we should now consider other investigative methods, such as US evaluation under weight-bearing conditions. Further studies designed to examine quantitative and qualitative changes in the IFP and their impact on AKP with a larger sample size are needed.

## Conclusion

A smaller change in IFP thickness after ACL reconstruction using the BTB technique was identified as a factor contributing to AKP on squatting. The results of this study may help in the evaluation of IFP dynamics after ACL reconstruction with the BTB technique.

## Data Availability

The datasets generated and/or analyzed during the current study are available from the corresponding author on reasonable request.
