# Correction: Relationship between the change in infrapatellar fat pad thickness assessed using ultrasonography and anterior knee pain on squatting after anterior cruciate ligament reconstruction

**DOI:** 10.1007/s10396-024-01457-5

**Published:** 2024-04-24

**Authors:** Ryo Shiraishi, Shinichiro Ueda

**Affiliations:** 1 Rokuto Orthopedic Surgery Az, 46 Onoyama-cho, Naha City, Okinawa 900-0026 Japan; 2 Department of Clinical Research and Quality Management, Graduate School of Medicine, University of The Ryukyus, 207 Uehara, Nishihara-cho, Okinawa, 903-0215 Japan; 3 Present Address: Department of Rehabilitation Therapy, Chuzan Hospital, 6-2-1 Matsumoto, Okinawa City, Okinawa 904-2151 Japan

**Correction: Journal of Medical Ultrasonics (2023) 50:237–243** 10.1007/s10396-023-01300-3

The article “Relationship between the change in infrapatellar fat pad thickness assessed using ultrasonography and anterior knee pain on squatting after anterior cruciate ligament reconstruction”, written by Ryo Shiraishi and Shinichiro Ueda, was originally published Online First without Open Access. After publication in volume 50, issue 2, pages 237–243 the author decided to opt for Open Choice and to make the article an Open Access publication. Therefore, the copyright of the article has been changed to © The Author(s) 2023 and the article is forthwith distributed under the terms of the Creative Commons Attribution 4.0 International License, which permits use, sharing, adaptation, distribution and reproduction in any medium or format, as long as you give appropriate credit to the original author(s) and the source, provide a link to the Creative Commons licence, and indicate if changes were made. The images or other third party material in this article are included in the article’s Creative Commons licence, unless indicated otherwise in a credit line to the material. If material is not included in the article’s Creative Commons licence and your intended use is not permitted by statutory regulation or exceeds the permitted use, you will need to obtain permission directly from the copyright holder. To view a copy of this licence, visit http://creativecommons.org/licenses/by/4.0.

The original article has been corrected.

